# Heterogeneity of treatment effect by baseline risk of mortality in critically ill patients: re-analysis of three recent sepsis and ARDS randomised controlled trials

**DOI:** 10.1186/s13054-019-2446-1

**Published:** 2019-05-03

**Authors:** Shalini Santhakumaran, Anthony Gordon, A. Toby Prevost, Cecilia O’Kane, Daniel F. McAuley, Manu Shankar-Hari

**Affiliations:** 1 Imperial Clinical Trials Unit, School of Public Health, Imperial College London, London, W12 7RH UK; 2 Section of Anaesthetics, Pain Medicine and Intensive Care, Imperial College London, London, W2 1NY UK; 3 Centre for Experimental Medicine, Wellcome-Wolfson Institute for Experimental Medicine, Belfast, BT9 7AE UK; 4 Regional Intensive Care Unit, Royal Victoria Hospital, Belfast, BT12 6BA UK; 5 Department of Intensive Care Medicine, St Thomas’ Hospital, Guy’s and St Thomas’ NHS Foundation Trust , Westminster Bridge Road, London, SE1 7EH UK; 6 Peter Gorer Department of Immunobiology, School of Immunology & Microbial Sciences, King’s College London, London, SE1 9RT UK

**Keywords:** Sepsis, acute respiratory distress syndrome, Models, statistical, Randomisation, Risk, Study design

## Abstract

**Background:**

Randomised controlled trials (RCTs) enrolling patients with sepsis or acute respiratory distress syndrome (ARDS) generate heterogeneous trial populations. Non-random variation in the treatment effect of an intervention due to differences in the baseline risk of death between patients in a population represents one form of heterogeneity of treatment effect (HTE). We assessed whether HTE in two sepsis and one ARDS RCTs could explain indeterminate trial results and inform future trial design.

**Methods:**

We assessed HTE for vasopressin, hydrocortisone and levosimendan in sepsis and simvastatin in ARDS patients, on 28-day mortality, using the total Acute Physiology And Chronic Health Evaluation II (APACHE II) score as the baseline risk measurement, comparing above (high) and below (low) the median score. Secondary risk measures were the acute physiology component of APACHE II and predicted risk of mortality using the APACHE II score. HTE was quantified both in additive (difference in risk difference (RD)) and multiplicative (ratio of relative risks (RR)) scales using estimated treatment differences from a logistic regression model with treatment risk as the interaction term.

**Results:**

The ratio of the odds of death in the highest APACHE II quartile was 4.9 to 7.4 times compared to the lowest quartile, across the three trials. We did not observe HTE for vasopressin, hydrocortisone and levosimendan in the two sepsis trials. In the HARP-2 trial, simvastatin reduced mortality in the low APACHE II group and increased mortality in the high APACHE II group (difference in RD = 0.34 (0.12, 0.55) (*p* = 0.02); ratio of RR 3.57 (1.77, 7.17) (*p* < 0.001). The HTE patterns were inconsistent across the secondary risk measures. The sensitivity analyses of HTE effects for vasopressin, hydrocortisone and levosimendan were consistent with the main analyses and attenuated for simvastatin.

**Conclusions:**

We assessed HTE in three recent ICU RCTs, using multivariable baseline risk of death models. There was considerable within-trial variation in the baseline risk of death. We observed potential HTE for simvastatin in ARDS, but no evidence of HTE for vasopressin, hydrocortisone or levosimendan in the two sepsis trials. Our findings could be explained either by true lack of HTE (no benefit of vasopressin, hydrocortisone or levosimendan vs comparator for any patient subgroups) or by lack of power to detect HTE. Our results require validation using similar trial databases.

**Electronic supplementary material:**

The online version of this article (10.1186/s13054-019-2446-1) contains supplementary material, which is available to authorized users.

## Background

Non-random variation in the treatment effect of an intervention due to differences in the baseline risk of death between patients in a population represents one form of heterogeneity of treatment effect (HTE) [1, 2]. In critical care settings, sepsis [3] and acute respiratory distress syndrome (ARDS) [4] are acute illnesses with significant clinical and biological heterogeneity [5–8]. Therefore, it is possible that even in randomised controlled trials (RCTs) which enrol patients that meet specific sepsis or ARDS eligibility criteria, there may still be heterogeneity in the trial populations. This heterogeneity occurs both within a trial and between trials [9]. The resulting variation in risk of outcomes may result in clinically important HTE in such trial populations. This heterogeneity is one possible explanation for indeterminate results in sepsis and ARDS RCT [9, 10]. We use the term *indeterminate* to illustrate that statistically non-significant results of two-tailed tests suggest uncertainty in results, as opposed to proof of no difference between treatments, implied by the term *negative* [11].

Recently, Iwashyna and colleagues simulated RCTs using observational cohort data and highlighted that the magnitude of HTE may be such that the average benefit (or harm) from the tested treatment in critical care RCTs may not be valid for an individual patient meeting the trial eligibility criteria [10]. Therefore, exploring HTE with data from completed RCTs where the intervention showed no effect in the overall population, aside from explaining the RCT results, could also inform future trial design and trial efficiency by targeting a trial population defined by a specific baseline measure associated either with the highest treatment benefit or with treatment response (enrichment) [9, 12].

In this context, we explored the presence of HTE for vasopressin and hydrocortisone in the VANISH trial [13], for levosimendan in the LeoPARDS trial [14] and for simvastatin in the HARP-2 trial [15]. We hypothesised that the individual patient’s baseline risk of death modifies the direction and magnitude of the treatment effects of vasopressin [13], hydrocortisone [13], levosimendan [14] and simvastatin [15] within these RCTs. A number of recent studies support our hypothesis. The treatment effect of vasopressin differed with severity of septic shock in a previous RCT [16]. The treatment effect of hydrocortisone differed between trials [17], with potential benefit seen in trials with higher control group mortality [18–20]. The treatment effect of simvastatin differed between ARDS sub-phenotypes [21] and potentially with illness severity in critically ill patients [22].

Our overall aim was to assess whether the individual patient’s baseline risk of death modifies the treatment effect of an intervention (HTE). The Acute Physiology And Chronic Health Evaluation II (APACHE II) model has been proposed as a potential model for HTE evaluation [10, 23, 24]. We assessed HTE using the APACHE II score [24] as the primary measure of baseline risk, and two secondary measures based on the APACHE II model: the APACHE II physiology score (APS-APII), and the APACHE II calculated risk of death as originally proposed by Knauss and colleagues (R) [24]. The rationale for using the APS-APII was that the total APACHE II score consists of non-modifiable risk of death from age and comorbidity, but the physiological derangement most likely mediates the treatment effect to outcome relationship [25]. Variation in the absolute risk difference may occur even if the relative effect of the treatment is the same, whilst the relative risk associated with the treatment may also vary. Therefore, we examined absolute and relative measures of heterogeneity. We also investigated whether any HTE could be driven by adverse events, as low-risk patients may have similar exposure to treatment-related harms to the high-risk patients, but not to the benefits, resulting in a net harm signal [10]. Furthermore, irrespective of whether the treatment effects of interventions varied or remained constant over the range of baseline risk, HTE may manifest due to differences in treatment-related adverse events over the range of baseline risk.

## Methods

### Study approvals and RCT datasets

We obtained ethics approval for this study (18/LO/1079). VANISH was a 2 × 2 factorial, double-blind, RCT in adult patients with sepsis who required vasopressors, in 18 general adult intensive care units (ICUs) in the United Kingdom (UK) [13]. In the VANISH trial [13], patients were randomly allocated to vasopressin and hydrocortisone (*n* = 101), vasopressin and placebo (*n* = 104), norepinephrine and hydrocortisone (*n* = 101) or norepinephrine and placebo (*n* = 103). Patients only received the second study drug (hydrocortisone/placebo) if the maximum infusion of the first study drug (vasopressin/norepinephrine) had been reached. Therefore, in the hydrocortisone analysis, only participants who received the study drug were included (hydrocortisone *n* = 148, placebo *n* = 148); all remaining analyses are intention-to-treat. The 28-day mortality was 63/204 (30.9%) of patients in the vasopressin group and 56/204 (27.5%) patients in the norepinephrine group (difference = 3.4% [95% CI, − 5.4–12.3%]) [13]. LeoPARDS was a two-arm parallel group, double-blind, placebo-controlled RCT in adult patients with sepsis who required vasopressors, in 34 ICUs in the UK [13]. In LeoPARDS trial [14], patients were randomised to receive either levosimendan (*n* = 258) or placebo (*n* = 257) over 24 h in addition to standard care. The 28-day mortality was 89/258 (34.5%) in the levosimendan group and 79/256 (30.9%) in the placebo group (difference = 3.6% [95% CI, − 4.5–11.7%]) [14]. HARP-2 was a two-arm parallel group, double-blind, placebo-controlled RCT in adult patients within 48 h after the onset of ARDS in 40 ICUs in the UK and Ireland [15]. In the HARP-2 trial [15], patients were randomised to receive either once-daily simvastatin or identical placebo tablets enterally for up to 28 days. The 28-day mortality was 57/259 (22.0%) in the simvastatin group and 75/280 (26.8%) in the placebo group (risk ratio = 0.8 [95% CI, 0.6 to 1.1]) [15].**]**

## Statistics

The primary analysis examined HTE for 28-day mortality with APACHE II score as the measure of baseline risk, comparing treatment effect in patients above (high) and below (low) the overall median score of 25. As secondary analyses, we examined two other baseline risk measures, APS-APII and *R*. Distributions of these baseline risk measures and mortality were described with histograms, and the discriminatory performance was assessed using the area under the receiver operating characteristic curve (AUC). We estimated the extreme quartile odds ratio (EQuOR, the ratio of the odds of death in the highest vs. lowest quartile for risk) as an estimate of how the risk of death varies between patients in the same trial [26]. Forest plots illustrated the absolute risk difference (RD) and relative risk (RR) for 28-day mortality by treatment group comparing high and low APACHE II groups. HTE was quantified on both the absolute and relative scales via additive and multiplicative interactions respectively. The difference in the RD and associated 95% confidence interval (CI) was estimated assuming a linear model for the probability of death, with treatment, a binary indicator for APACHE II subgroup, and the interaction between them as covariates, using robust standard errors. The ratio of the RR and 95% CI was estimated assuming a log-binomial model with the same covariates. We then investigated heterogeneity of harms using forest plots by APACHE II subgroup similar to the primary analysis. Interactions were not estimated for heterogeneity of harms due to the low number of events. For the HARP-2 trial, only the primary baseline risk measure of the total APACHE II score was available.

### Sensitivity analyses

Four sensitivity analyses for the main baseline risk measure (APACHE II score) were performed. First, we used hospital mortality as the outcome instead of at 28 days, as APACHE II score was originally devised as a prediction tool for hospital mortality. Second, we investigated the potential impact of missing data on the results. In the VANISH trial, there were 47 patients who had at least one element of the acute physiology score missing, and 61 patients in the LeoPARDS trial. In the main analysis, normal scores were assumed for these elements, as for the main trial [13, 14]. In the HARP-2 trial, 66 patients had missing total APACHE II scores and were omitted from the main analysis but displayed in the forest plot. Missingness occurred pre-randomisation and hence is independent of the treatment effect but may affect the precision of the results. In the sensitivity analysis, we assumed patients with missing data were (i) equally likely to be in the high-risk group as those with complete data, (ii) 10% more likely and (iii) 10% less likely. APACHE II category was imputed 20 times under these assumptions, and the difference in RD and ratio of RR computed as for the main analysis, combining results across imputations using Rubin’s rules. A third sensitivity analysis was performed by recalibrating the APACHE II risk prediction using the whole RCT cohort, as internally developed risk models using both treatment arms are preferred to models developed on the control population, as highlighted by Burke et al. [27]. A logistic regression model for 28-day mortality was constructed with the following covariates: APACHE II points from each acute physiology component, age points, chronic health points, post-emergency surgery and diagnostic category weight. The resulting prediction was used as a measure of baseline risk, assessing HTE as in the main analysis. To avoid spurious associations from categorisation of APACHE II score [28], we performed a fourth sensitivity analysis, by treating APACHE II as a continuous variable in a logistic regression model. Relative HTE was quantified by the interaction between APACHE II score and treatment, expressed as a ratio of odds ratios. Additive HTE was illustrated by plotting the estimated absolute difference in mortality between treatment groups across the range of APACHE II.

## Results

### Baseline risk of 28-day mortality

**Fig. 1:**
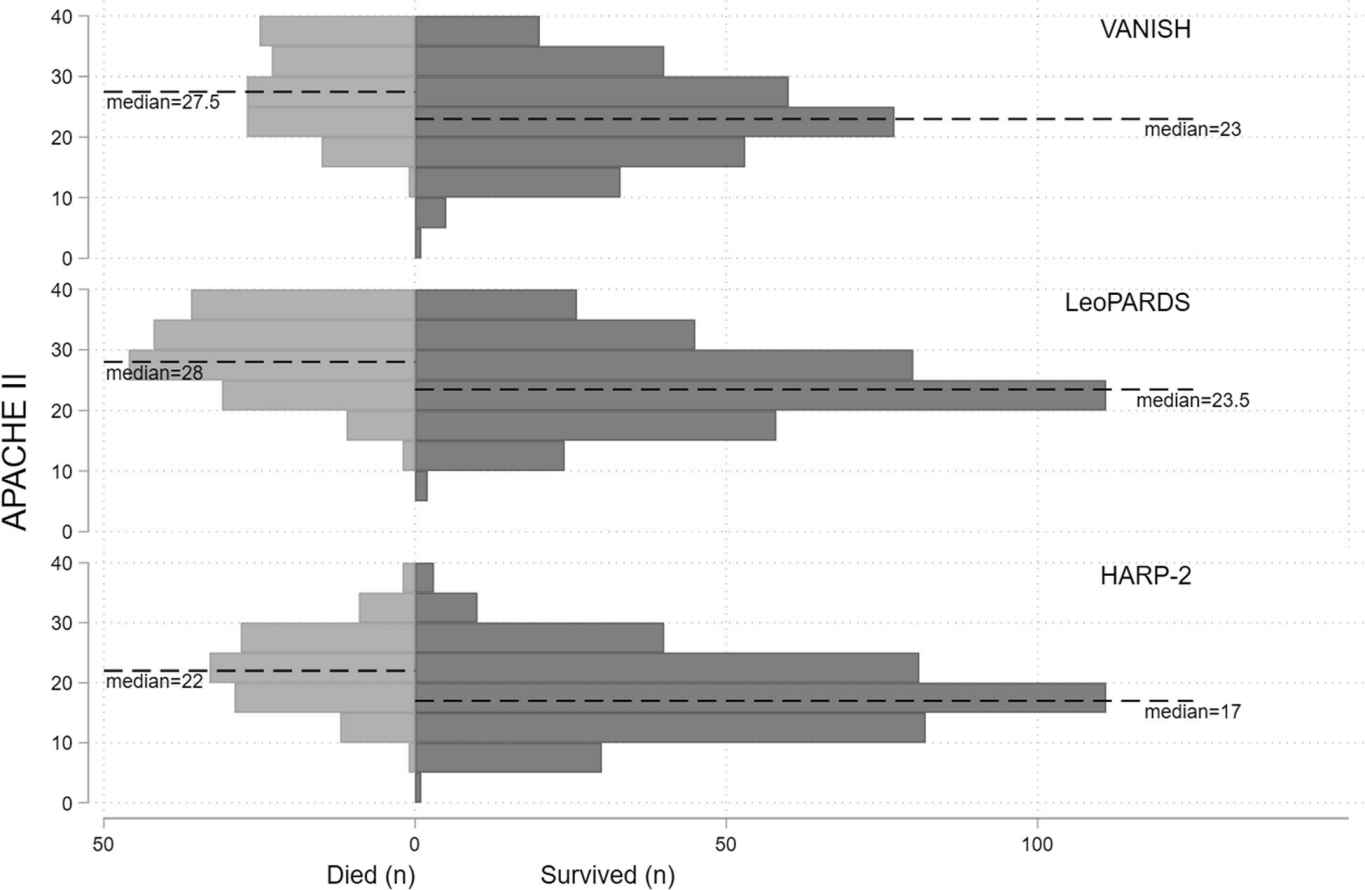
Histogram showing distribution of APACHE II score by 28-day mortality and trial

The 28-day mortality, between the intervention and control arms, in the VANISH, LeoPARDS and HARP-2 trials was not significantly different (Table 1). The illness severity (using the total APACHE II score) was lower in the HARP-2 trial, compared to those in the VANISH and LeoPARDS trials (Fig. 1). The EQuOR highlighted significant heterogeneity of risk of death in all three RCTs for all three risk measures.

**Table 1:**
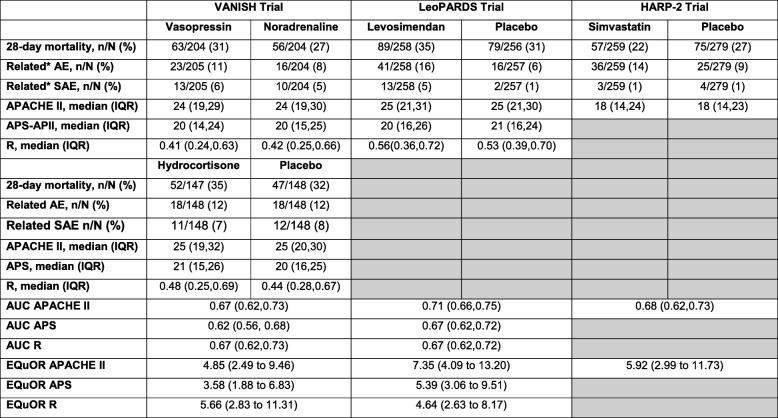
Trial level summary characteristics

Shaded regions in the HARP-2 trial represent lack of raw data to derive APS-APII score or *R*

*IQR* interquartile range, *AUC* area under the receiver operating characteristic curve, *EQuOR* extreme quartile odds ratio, *(S)AE* (serious) adverse events, *APACHE II* Acute Physiology And Chronic Health Evaluation II, *APS-APII* Acute Physiology Score from APACHE II, *R* risk of death calculated from APACHE II

### VANISH trial HTE assessment

**Fig. 2:**
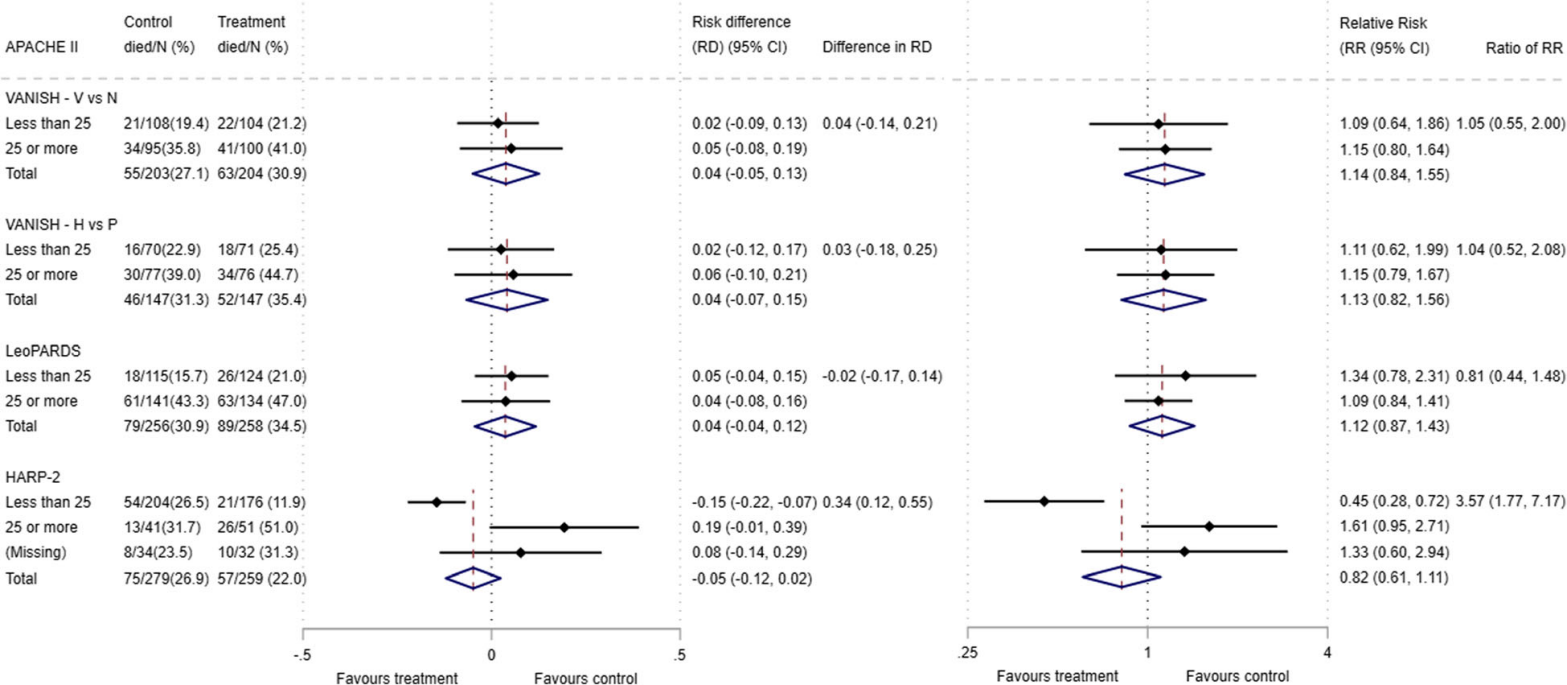
Forest plots for the risk difference and risk ratio comparing 28-day mortality in treatment and control, by trial and APACHE II low and high groups

**Fig. 3:**
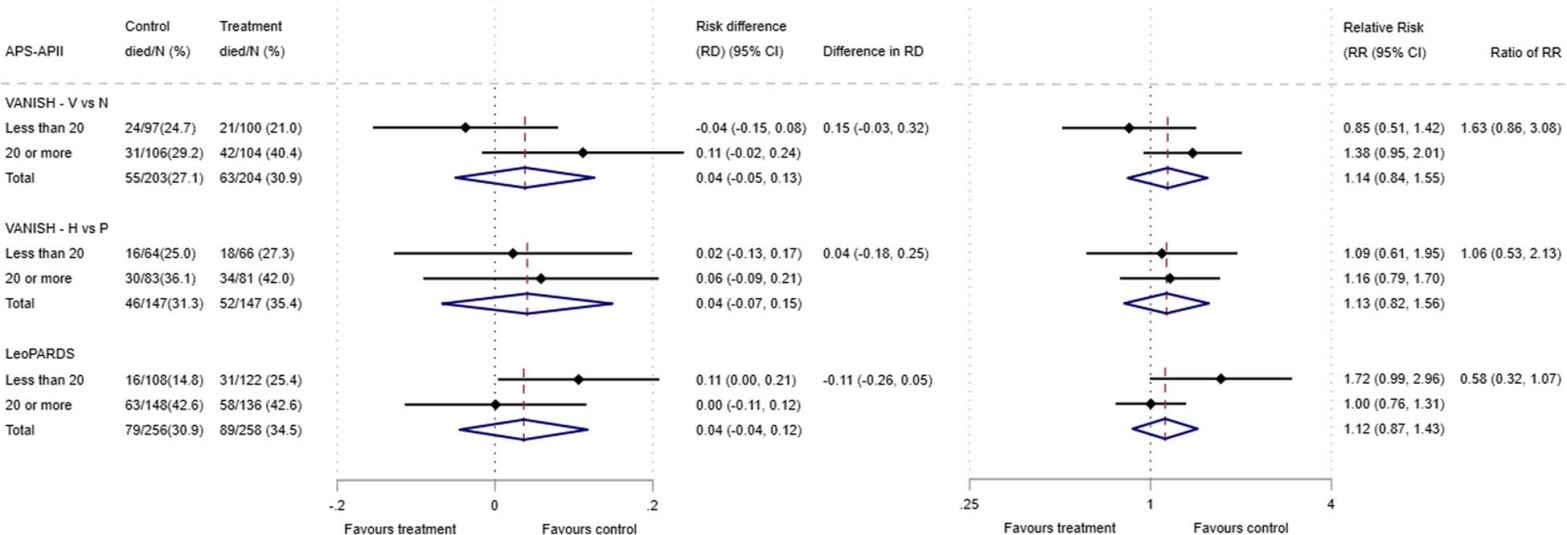
Forest plots for the risk difference and risk ratio comparing 28-day mortality in treatment and control, by trial and APS-AP-II low and high groups

**Fig. 4:**
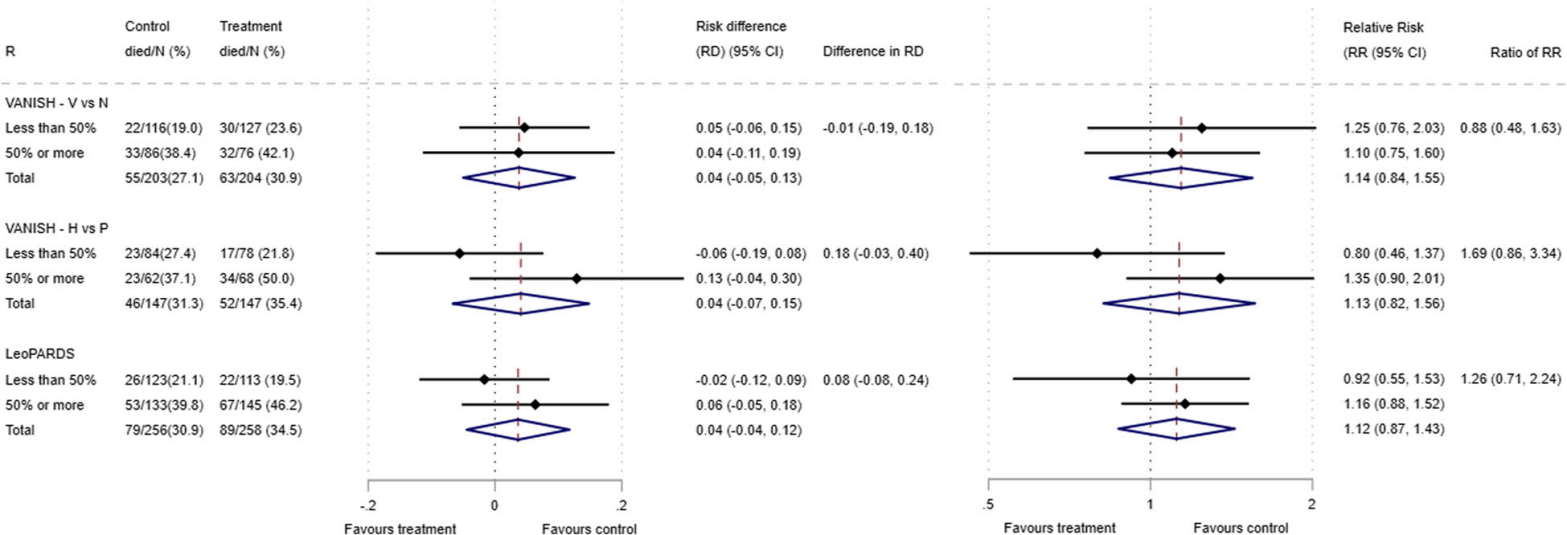
Forest plots for the risk difference and risk ratio comparing 28-day mortality in treatment and control, by trial and *R* low and high groups

The 28-day mortality increased in the vasopressin and in the norepinephrine group, with increasing baseline risk measures (Fig. 1). For the primary analysis with APACHE II score as baseline risk of death measure, there was no evidence of HTE for vasopressin in either absolute terms (risk difference for low APACHE II 0.02 (− 0.09, 0.13) and high APACHE II 0.05 (− 0.08, 0.19); difference in risk difference 0.04 (− 0.14, 0.21)) or relative terms (relative risk for low APACHE II 1.09 (0.64, 1.86) and high APACHE II 1.15 (0.08, 1.64); ratio of relative risk 1.05 (0.55, 2.00)) (Fig. 2). For the secondary risk measures, the estimates of HTE for vasopressin were larger with wider CI for APS-APII (Fig. 3) and smaller in magnitude for *R* (Fig. 4).

For the primary analysis with APACHE II score as baseline risk of death measure, there was no evidence of HTE for hydrocortisone in either absolute terms (risk difference for low APACHE II 0.02 (− 0.12, 0.17) and high APACHE II 0.06 (− 0.10, 0.21); difference in risk difference 0.03 (− 0.18, 0.25) or relative terms (relative risk for low APACHE II 1.11 (0.62, 1.99) and high APACHE II 1.15 (0.79, 1.67); ratio of relative risk 1.04 (0.52, 2.08)). For the secondary risk measures, the estimates of HTE for hydrocortisone were similar for APS-APII (Fig. 3) and larger in magnitude for *R* (Fig. 4).

### LeoPARDS trial HTE assessment

The 28-day mortality increased in the levosimendan and in the placebo group, with increasing baseline risk measures (Fig. 1). For the primary analysis with APACHE II score as baseline risk of death measure, there was no evidence of HTE for levosimendan in either absolute terms (risk difference for low APACHE II 0.05 (− 0.04, 0.15) and high APACHE II 0.04 (− 0.08, 0.16); difference in risk difference − 0.02 (− 0.17 to 0.14)) or relative terms (relative risk for low APACHE II 1.34 (0.78, 2.31) and high APACHE II 1.09 (0.84, 1.41); ratio of relative risk 0.81 (0.44 to 1.48)) (Fig. 2). For the secondary risk measures, the estimates of HTE for levosimendan were larger for APS-APII (Fig. 3) and in the opposite direction for *R* (Fig. 4).

### HARP-2 trial HTE assessment

The 28-day mortality increased in the simvastatin group and in the standard care group, with increasing baseline risk measures (Fig. 1). For the primary analysis with APACHE II score as baseline risk of death measure, we observed HTE for simvastatin in absolute terms (risk difference for low APACHE II − 0.15 (− 0.22, − 0.07) and high APACHE II 0.19 (− 0.01, 0.39); difference in risk difference 0.34 (0.12, 0.55) (*p* = 0.02)) and in relative terms (relative risk for low APACHE II 0.45 (0.28, 0.72) and high APACHE II 1.61 (0.95, 2.71); ratio of relative risk 3.57 (1.77 to 7.17)). Simvastatin reduced mortality in the low APACHE II group and increased mortality in the high APACHE II group (Fig. 2). As raw data APACHE II score data were not available, we have not reported any secondary risk measures for the HARP-2 trial.

### Serious adverse events and baseline risk

We plotted the proportions of serious adverse events by low and high APACHE II groups in each trial, to explore whether the pattern of adverse event distribution could explain any HTE in mortality. In all three RCTs, both in the intervention and control trial arms, there was no pattern in serious adverse events that could explain HTE in mortality (Additional file 1: Figure S1).

### Sensitivity analyses

Results from sensitivity analyses were consistent with the main analyses for the VANISH and LeoPARDS trials (Additional file 1: Table S1*,* Table S2, Figure S2*,* Figure S3 and Figure S4). HTE effects were attenuated in the sensitivity analyses for the HARP-2 trial under different assumptions for the missing data (e.g. ratio of relative risk was 2.86 (1.47, 5.57) when we assumed that patients with missing APACHE II data were more likely to be high risk; all other results were less attenuated Additional file 1: Table S1). Differences were also smaller when hospital mortality was used as the outcome (difference in risk difference 0.25 (0.03, 0.48); ratio of RR 2.34 (1.31, 4.18), Additional file 1: Figure S1) and when HTE was assessed across the continuous range of APACHE II score (ratio of odds ratio for a 5-point increase in APACHE II 1.33 (0.93, 1.90) Additional file 1: Table S2 and Figure S4).

## Discussion

We assessed whether HTE could contribute to the indeterminate results in three recent ICU RCTs, using multivariable baseline risk of death models, which included well-established risk factors for acute mortality for sepsis and ARDS as covariates. There was considerable within-trial variation in the baseline risk of death in all three RCTs. We did not observe HTE for vasopressin, hydrocortisone and levosimendan in the two sepsis trials, though there was evidence of differential treatment effect in the HARP-2 trial for ARDS with low risk of death sub-population benefitting the most. We observed that detection of HTE in RCTs may be influenced by the baseline risk model specification, as illustrated by differences in HTE effects seen with different models reported using the LeoPARDS trial data.

### Explanation of key findings

There are a number of possible reasons why we did not observe HTE consistently in all our analyses. All three trials we assessed have many features of explanatory trials [29], which by their design limit HTE in comparison to pragmatic trials, such as through narrower eligibility criteria, intensity of follow-up and non-mortality primary outcomes. Therefore, demonstrable HTE is less likely in these trials, though its evaluation remains important. Our findings may be true in that HTE may be less marked in sepsis and ARDS as mortality may be driven by non-modifiable risk factors such as older age and presence of comorbid conditions, alongside illness attributable risk, generating many “minimal contributory causes” of mortality [30], when compared to illnesses such as retroviral disease [31]. It could be that the effects of treatments we assessed on mortality are both small and with limited variability across baseline risk of death resulting in minimal HTE. Another explanation for not observing HTE may be that there is no true treatment effect difference between subgroups enriched on prognosis with APACHE II score. Given the sample size in the trials assessed, we may only have power to detect large interaction effects, which requires either a large treatment effect in one or more subgroups, opposing treatment effects between subgroups or differential adverse event risk between subgroups.

### Comparison to published literature

A key comparison to consider is the contrasting results with RCT simulations by Iwashyna and colleagues [10]. Their simulations assumed that the trial participants’ odds of 30-day mortality will be influenced by the severity of acute respiratory failure, comorbid conditions, the treatment’s reduction in mortality from the primary illness and the treatment’s fatal adverse effect rates. Furthermore, Iwashyna and colleagues assumed constant relative treatment effects, constant harms and mortality patterns predicted by their simulation model. We used 28-day mortality, for our primary analysis. We considered baseline risk of death as a function of acute illness severity using the total APACHE II score, which is in line with the conceptual arguments put forward by Kent and colleagues [1] that HTE emerges from the risk of outcome (28-day mortality in our study), the risk of treatment-related harm and direct treatment-effect modification. Importantly, the data in our trials do not follow the constant relative treatment effects, constant harms and therefore the mortality patterns described by Iwashyna and colleagues [10].

Recently, Semler and colleagues reported a pragmatic, cluster-randomised, multiple-crossover trial of saline versus balance crystalloids in critically ill patients, with no difference in primary outcome of major adverse kidney events within 30 days (MAKE30), a composite of in-hospital death, new receipt of renal-replacement therapy and persistent renal dysfunction [32], but reported presence of HTE for this outcome with a multivariable model specifically calibrated for this outcome [33]. In contrast, our analytic strategy ascertained whether the observed treatment effect differed by the pre-randomisation baseline risk of death multivariable model (APACHE II score), which helped us to compare multiple treatments in critically ill patients with sepsis or ARDS.

### Strengths and weakness

We explored heterogeneity in absolute and in relative treatment effects, in sepsis and ARDS, for four different treatments and using three different measures of baseline risk. The primary baseline risk measure, APACHE II, is an established, validated predictor of mortality in this population. Two variations on this measure were investigated to check the consistency of the results, along with several sensitivity analyses. We used a composite risk score (APACHE II) for its superior performance for baseline risk estimation, as highlighted by Kent and colleagues [1] and a recommendation for future studies on HTE assessment [10]. There were insufficient numbers to examine HTE across smaller groups (e.g. quartiles). None of the RCTs included in this study had 28-day mortality as the primary outcome; it is possible that we were underpowered to detect HTE. The primary outcomes were not suitable for HTE analysis as they were continuous rather than binary and without an appropriate baseline measure, though the existing HTE framework could be adapted for some continuous outcomes, such as change from baseline organ dysfunction.

### Implications of research

Aside from the ARDS or sepsis illness characteristics, it is likely that biological mechanisms determining differences in treatment effect will vary with the intervention tested. Therefore, using a generic physiology-based multivariable model such as APACHE II with biomarkers that provide both prognostic and predictive enrichment or intervention-specific predictive enrichment coupled may be a better approach to defining a study population. For example, an ARDS sub-population with greater inflammation and higher mortality was more likely to benefit from simvastatin [21, 34], and aside from severity of septic shock, the treatment effect of vasopressin was associated with biological differences within the trial population [16, 35]. Similarly, biomarkers derived from whole blood transcriptomics could help enrich septic shock patients for corticosteroid therapy [36–38]. It is plausible that when HTE is assessed for an intervention using data from a single trial, we are unlikely to detect it unless HTE effects are large. This generates an argument to assess HTE using trials of the similar treatment-condition combination or of the same condition and broader group of treatments with similar enough mechanism of treatment effect, or consider intervention-specific multivariable models. As suggested by Iwashyna and colleagues, perhaps HTE assessment should form part of a priori analyses plans in future clinical trials. As HTE is about the variation in effectiveness, standardising the baseline risk measure between RCTs, including HTE assessment as a priori analyses, ensuring that the outcome used in HTE analyses is patient-centered (such as mortality) and incorporating the proposals within the Core Outcome Measures in Effectiveness Trials guidelines will enable pooling of HTE analysis across future trials [39].

## Conclusions

We assessed HTE in three recent ICU RCTs, using multivariable baseline risk of death models. There was considerable within-trial variation in the baseline risk of death. We observed potential HTE for simvastatin in ARDS, but no evidence of HTE for vasopressin, hydrocortisone or levosimendan in the two sepsis trials. Our findings could be explained either by true lack of HTE (no benefit of vasopressin, hydrocortisone or levosimendan vs comparator for any patient subgroups) or by lack of power to detect HTE. Our results require validation using similar trial databases.

## Additional file

Additional file 1: **Table S1.** Results from multiple imputation analysis; for patients with missing APACHE II, we assumed the proportion in the high-risk category (APACHE II ≥ 25) was either the same as the trial participants with complete data, 10% higher or 10% lower. **Table S2.** Treatment-risk interaction using continuous APACHE II from logistic regression analysis of 28-day mortality. **Figure S1.** Forest plots for the risk difference and risk ratio comparing related serious adverse events in treatment and control, by trial and APACHE II subgroup. **Figure S2.** Forest plots for the risk difference and risk ratio comparing hospital mortality in treatment and control, by trial and APACHE II subgroup. **Figure S3.** Forest plots for the risk difference and risk ratio comparing related serious adverse events in treatment and control, by trial and APACHE II subgroup. **Figure S4.** HTE assessment for APACHE II score as a continuous variable. Figures show the estimated treatment effect with 95% confidence interval bands from regression models for 28-day mortality including a treatment × APACHE II score interaction for **Figure S4A:** VANISH Vasopressin; **Figure S4B:** VANISH Hydrocortisone; **Figure S4C:** LeoPARDS and **Figure S4D:** HARP-2. (DOCX 577 kb)
