# Supplementary material for: Heterogeneity of treatment effect by baseline risk of mortality in critically ill patients: re-analysis of three recent sepsis and ARDS randomised controlled trials
Source: Crit Care. 2019 May 3;23:156. doi: 10.1186/s13054-019-2446-1 (PMC6500045; doi:10.1186/s13054-019-2446-1)
Supplement: Supplementary file 1 — Table S1. Results from multiple imputation analysis; for patients with missing APACHE II, we assumed the proportion in the high-risk category (APACHE II ≥ 25) was either the same as the trial participants with complete data, 10% higher or 10% lower. Table S2. Treatment-risk interaction using continuous APACHE II from logistic regression analysis of 28-day mortality. Figure S1. Forest plots for the risk difference and risk ratio comparing related serious adverse events in treatment and control, by trial and APACHE II subgroup. Figure S2. Forest plots for the risk difference and risk ratio comparing hospital mortality in treatment and control, by trial and APACHE II subgroup. Figure S3. Forest plots for the risk difference and risk ratio comparing related serious adverse events in treatment and control, by trial and APACHE II subgroup. Figure S4. HTE assessment for APACHE II score as a continuous variable. Figures show the estimated treatment effect with 95% confidence interval bands from regression models for 28-day mortality including a treatment × APACHE II score interaction for Figure S4A: VANISH Vasopressin; Figure S4B: VANISH Hydrocortisone; Figure S4C: LeoPARDS and Figure S4D: HARP-2. (DOCX 577 kb) [file 13054_2019_2446_MOESM1_ESM.docx]

**Online only supplementary material**

**Santhakumaran S et al.** **Heterogeneity of Treatment Effect by Baseline Risk of Mortality in Critically Ill Patients: Re-analysis of three recent Sepsis and ARDS Randomised Controlled Trials**.

**Supplementary Tables captions**

**Table S1:** Results from multiple imputation analysis; for patients with missing APACHE-II, we assumed the proportion in the high-risk category (APACHE-II≥25) was either the same as the trial participants with complete data, 10% higher or 10% lower.

**Table S2:** Treatment-risk interaction using continuous APACHE II from logistic regression analysis of 28-day mortality

**Supplementary Figures captions**

**Figure S1:** Forest plots for the risk difference and risk ratio comparing related serious adverse events in treatment and control, by trial and APACHE-II subgroup

**Figure S2*:*** Forest plots for the risk difference and risk ratio comparing hospital mortality in treatment and control, by trial and APACHE-II subgroup

**Figure S3*:*** Forest plots for the risk difference and risk ratio comparing 28-day mortality in treatment and control, by trial and recalibrated APACHE-II subgroup

**Figure S4*:*** HTE assessment for APACHE II score as a continuous variable in a logistic regression model

Figures shows the estimated treatment effect with 95% confidence interval bands from regression models for 28-day mortality including a treatment x APACHE II score interaction for **eFig4A:** VANISH Vasopressin; **eFig4B:** VANISH Hydrocortisone; **eFig4C:** LeoPARDS and **eFig4D:** HARP-2.

**Table S1 Results from multiple imputation analysis; for patients with missing APACHE-II, we assumed the proportion in the high risk category (APACHE-II≥25) was either the same as the trial participants with complete data, 10% higher or 10% lower.**

| Trial, and assumption for missing APACHE-II | % APACHE-II≥25 | Difference in RD (95% CI) | Ratio of RR (95% CI) |
| --- | --- | --- | --- |
| VANISH V-N |  |  |  |
| Same as complete data | 50% | 0.03 (-0.16,0.21) | 1.02 (0.51, 2.04) |
| 10% higher | 60% | 0.03 (-0.16, 0.21) | 1.03 (0.51, 2.07) |
| 10% lower | 40% | 0.01 (-0.17, 0.20) | 0.96 (0.49, 1.90) |
| VANISH HC-P |  |  |  |
| Same as complete data | 50% | 0.03 (-0.19,0.25) | 1.02 (0.49, 2.14) |
| 10% higher | 60% | 0.03 (-0.20, 0.25) | 1.03 (0.48, 2.19) |
| 10% lower | 40% | 0.02 (-0.21, 0.25) | 0.99 (0.47, 2.09) |
| LeoPARDS |  |  |  |
| Same as complete data | 56% | -0.01 (-0.17, 0.17) | 0.89 (0.47, 1.69) |
| 10% higher | 66% | -0.01 (-0.17, 0.16) | 0.86 (0.45, 1.67) |
| 10% lower | 46% | -0.02 (-0.17, 0.17) | 0.89 (0.47, 1.69) |
| HARP-2 |  |  |  |
| Same as complete data | 19% | 0.31 (0.10, 0.52) | 2.99 (1.51, 5.90) |
| 10% higher | 29% | 0.30 (0.08, 0.52) | 2.96 (1.43, 6.10) |
| 10% lower | 18% | 0.30 (0.09, 0.51) | 2.86 (1.47, 5.57) |

**Table S2: Treatment-risk interaction using continuous APACHE II from logistic regression analysis of 28-day mortality**

| **Trial** | **Ratio of OR for a 5 point increase in APACHE II** | **95% confidence interval** |
| --- | --- | --- |
| **VANISH V-N** | **0.96** | **(0.71, 1.29)** |
| **VANISH HC-P** | **0.93** | **(0.67, 1.29)** |
| **LeoPARDS** | **1.00** | **(0.74, 1.34)** |
| **HARP-2** | **1.33** | **(0.93, 1.90)** |

**Figure S1 Forest plots for the risk difference and risk ratio comparing related serious adverse events in treatment and control, by trial and APACHE-II subgroup**
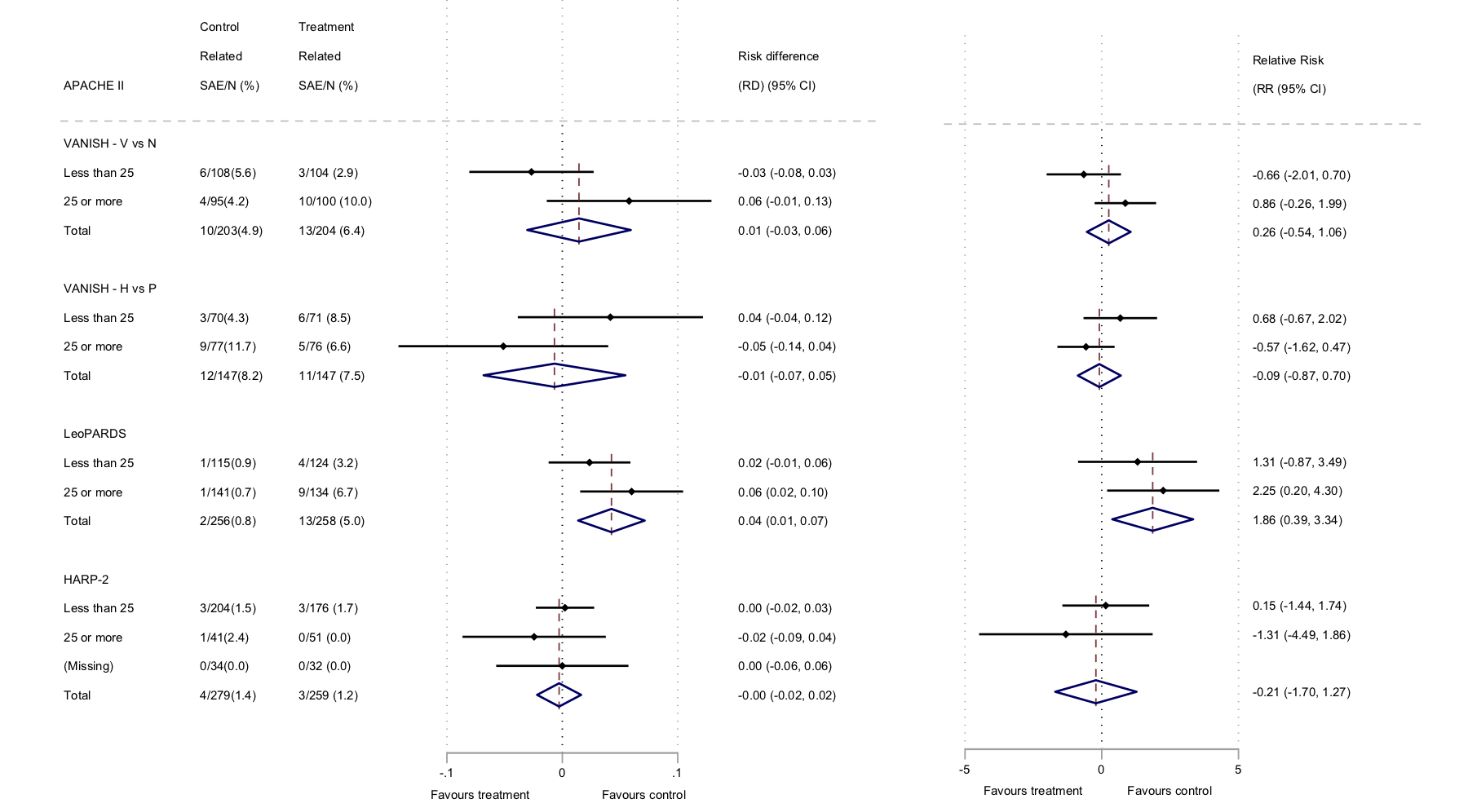


**Figure S2 Forest plots for the risk difference and risk ratio comparing hospital mortality in treatment and control, by trial and APACHE-II subgroup**

**
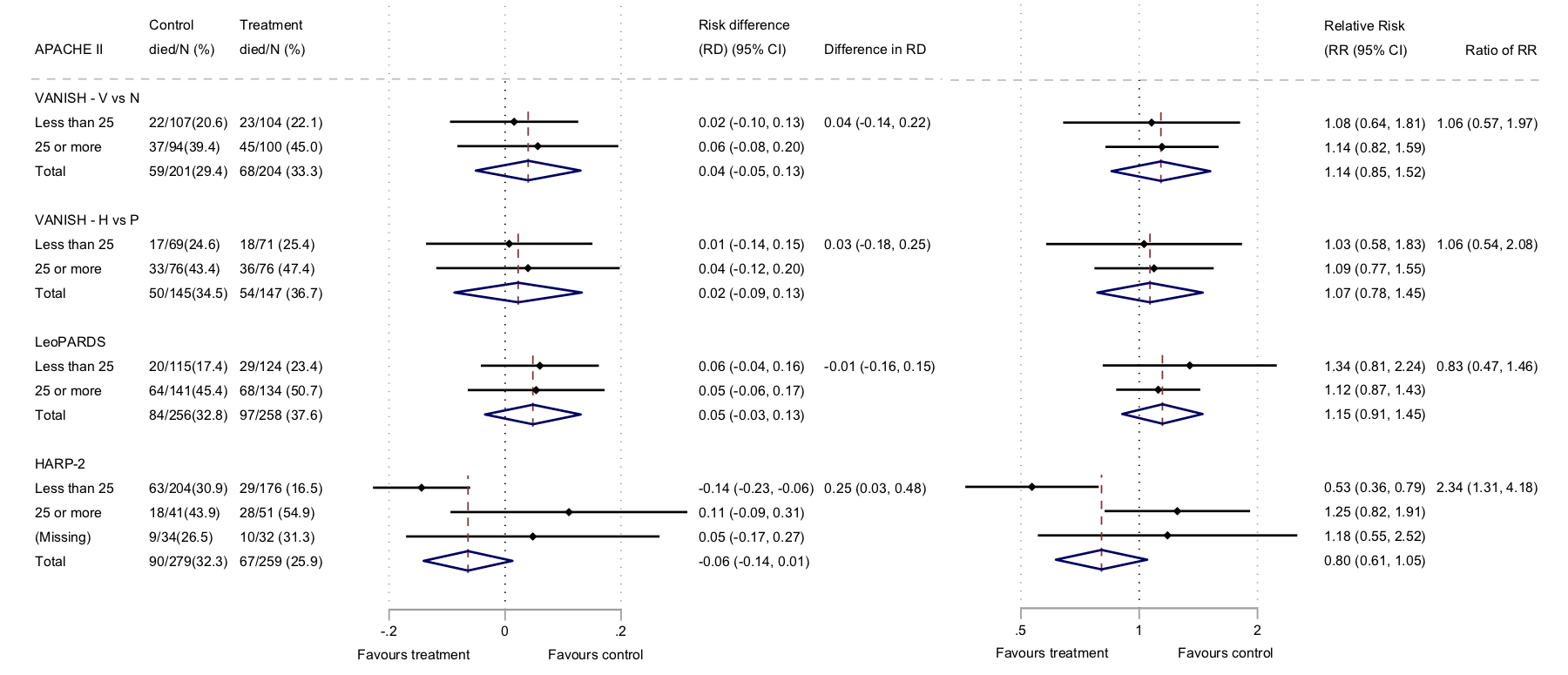
**

**Figure S3*:*** Forest plots for the risk difference and risk ratio comparing 28-day mortality in treatment and control, by trial and recalibrated APACHE-II subgroup

**Figure S4*:*** HTE assessment for APACHE II score as a continuous variable
